# Borax Cross-Linked Acrylamide-Grafted Starch Self-Healing Hydrogels

**DOI:** 10.1021/acs.biomac.4c01287

**Published:** 2024-11-25

**Authors:** Kai Lu, Xiaohong Lan, Rudy Folkersma, Vincent S. D. Voet, Katja Loos

**Affiliations:** † Macromolecular Chemistry and New Polymeric Materials, Zernike Institute for Advanced Materials, University of Groningen, Nijenborgh 3, 9747AG Groningen, The Netherlands; ‡ Circular Plastics, Academy Technology & Innovation, NHL Stenden University of Applied Sciences, Van Schaikweg 94, 7811 KL Emmen, The Netherlands

## Abstract

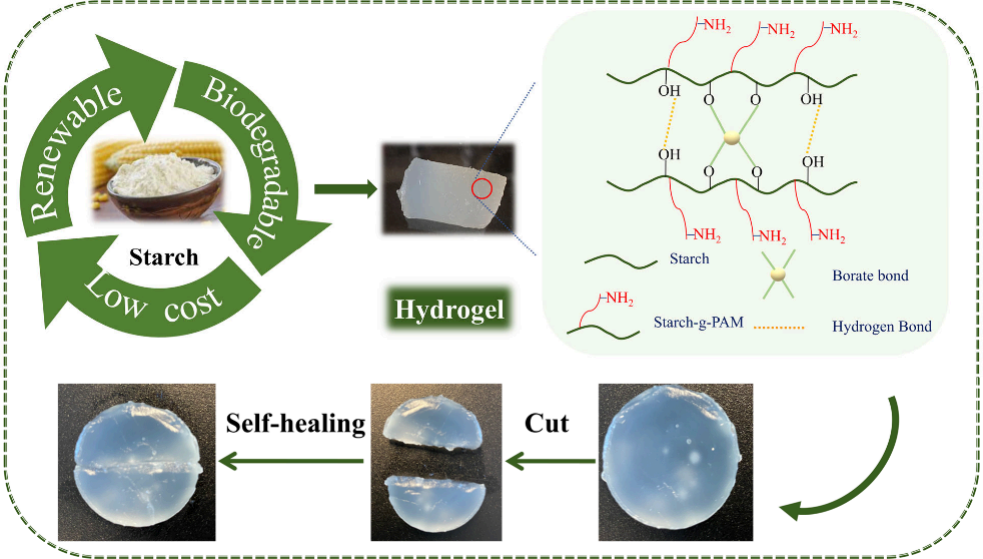

The biocompatibility and renewability of starch-based hydrogels have made them popular for applications across various sectors. Their tendency to incur damage after repeated use limits their effectiveness in practical applications. Improving the mechanical properties and self-healing of hydrogels simultaneously remains a challenge. This study introduces a new self-healing hydrogel, synthesized by grafting acrylamide onto starch using ceric ammonium nitrate (CAN) as an initiator, followed by borax cross-linking. We systematically examined how the starch-to-monomer ratio, borax concentration, and CAN concentration impact the grafting reactions and overall performance of the hydrogels. The addition of borax significantly reinforced the strength of the hydrogel; the maximum storage modulus increased by 1.8 times. Thanks to dynamic borate ester and hydrogen bonding, the hydrogel demonstrated remarkable recovery properties and responsiveness to temperature. We expect that the present research could broaden the application of starch-based hydrogels in agriculture, sensors, and wastewater treatment.

## 1 Introduction

Hydrogels consist of three-dimensional networks formed by the physical or chemical cross-linking of hydrophilic macromolecules. They possess a high capacity for water absorption, allowing them to retain substantial amounts of water without compromising their structure, even under pressure.^1,2^ Due to these properties, hydrogels are used across diverse fields, including food, architecture, pharmaceuticals, treatment of wastewater, and agriculture.^3−7^ Conventional hydrogels typically rely on petroleum-derived polymers, which are expensive, nonbiodegradable, and may pose environmental risks.^8,9^ This has led to growing interest in natural hydrogels made from cellulose, alginate, chitosan, lignin, and starch, valued for their biocompatibility, renewability, and nontoxicity.^10,11^

Starch, a primary energy source for various plants, is one of the most abundant natural polysaccharides, known for being renewable, biodegradable, and cost-effective.^12−15^ Additionally, each repeating unit in starch has multiple hydroxyl groups, making it highly suitable for chemical or physical cross-linking to create hydrogels.^16−18^ However, starch hydrogels generally have weak mechanical properties due to their single-network structure, requiring enhancement through grafting with functional monomers.^19−21^ Moreover, starch-based hydrogels typically lose mechanical integrity and cannot recover after damage, limiting their lifespan and practical applications.^22−24^ Improving the mechanical strength and self-healing properties of starch hydrogels is critical to boost their longevity and functionality.

Numerous approaches have been explored to develop hydrogels with self-healing capabilities and enhanced mechanical strength, often through the use of dual cross-linked networks (DCs) or the addition of dynamic covalent or noncovalent bonds.^25,26^ Noncovalent interactions in hydrogels primarily include hydrophobic interactions, electrostatic forces, host–guest interactions, ionic bonds, and hydrogen bonds.^27−31^ Hydrogel networks often incorporate dynamic covalent bonds such as those formed through Diels–Alder reactions, disulfide, imine, acylhydrazone, and borate ester bonds.^32−36^ Borax acts as a reversible cross-linker, dissociating into boric acid and borate ions (B(OH)_4_^–^) in water.^37^ Borate ions are capable of forming dynamic ester bonds with hydroxyl groups in starch, facilitating the development of self-healing hydrogels with enhanced mechanical strength.^38,39^ Additionally, these bonds are responsive to changes in pH and temperature, making the hydrogels stimuli-responsive.^40,41^ Qin et al. developed a hybrid dual cross-linked (DC) hydrogel based on starch/PVA/borax that demonstrated excellent stretchability, toughness, and self-healing properties. The presence of hydrogen bonds and dynamic borate ester bonds within the dual cross-linked network contributed to these characteristics.^42^ Siriporn et al. reported that incorporating borax enhanced the dimensional stability as well as the mechanical and thermal properties of hydrogels.^43^ Chen et al. developed double cross-linked (DC) starch-borax hydrogels that exhibited improved toughness and self-healing properties. They observed that adding borax significantly boosted the storage modulus of these hydrogels, achieving a *G*′max of approximately 2000 Pa.^19^ Furthermore, the mildly alkaline environment produced by acrylamide facilitates boronic esterification. Because borate ester bonds are pH-sensitive, at lower pH levels, B(OH)_4_^–^ is converted to B(OH)_3_, a weaker cross-linker, resulting in the formation of softer, more flexible gels.^44^ Based on these insights, we propose that using borax as a cross-linker in combination with acrylamide grafting onto starch could improve both the mechanical strength and self-healing ability of the hydrogel. To our knowledge, this type of starch-based hydrogel has not been previously reported.

In this research, we developed an innovative self-healing and thermally responsive hydrogel by grafting acrylamide onto starch, followed by cross-linking with borax. The grafting of acrylamide improved the hydrogel’s hydrophilicity. We extensively investigated the effects of starch-to-monomer ratio, borax concentration, and ceric ammonium nitrate (CAN) concentration on grafting reactions and the resulting hydrogel properties. This study analyzed the swelling behavior, along with rheological, microstructural, self-healing, thermosensitive, and thermal properties of the synthesized hydrogels. With enhanced mechanical and self-healing features, we suggest that this study expands the potential applications for starch-based hydrogels.

## 2 Materials and Methods

### 2.1 Materials

Corn starch (CS, product code S4126, amylose content 27%, moisture content ≤15.0%, pH 4.0–7.0), acrylamide (AM), borax (Na_2_B_4_O_7_·10H_2_O), ceric ammonium nitrate (CAN), ethanol, deuterated dimethyl sulfoxide (DMSO-*d*_6_, 99.9%) and deuterium oxide (D_2_O, 99.9%) were purchased from Sigma-Aldrich. Distilled water was used in the preparation of all the solutions. All chemicals were of analytical grade and used without further purification.

### 2.2 Preparation of Borax Cross-Linked Acrylamide-Grafted Starch Hydrogels

**Scheme 1:**
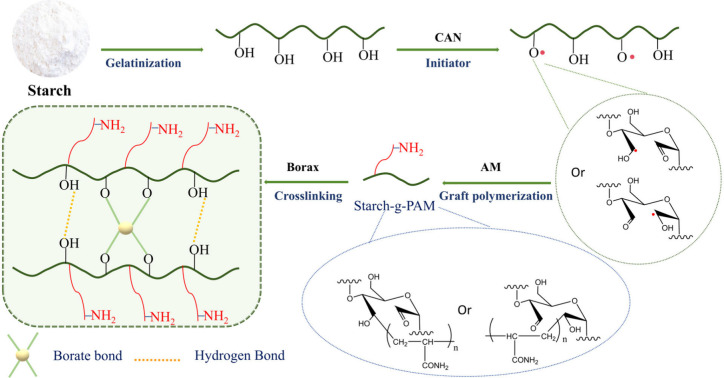
Method and Mechanism of Synthesis of Borax Cross-Linked Acrylamide-Grafted Starch Hydrogels

The hydrogels were synthesized following the procedure outlined in Scheme 1. A specific amount of starch was dissolved in deionized water while stirring at 80 °C for 30 min under a nitrogen atmosphere, producing a fully gelatinized starch solution. Following this, 5 mL of freshly prepared CAN solution was introduced at 60 °C for 10 min to initiate free radical formation on the starch, still under a nitrogen atmosphere. A fresh acrylamide (AM) solution was then added at 60 °C, along with a borax solution, and the reaction proceeded for 2 h in a nitrogen atmosphere. The final product was rinsed with distilled water and soaked in acetone to eliminate unreacted borax and ungrafted monomers. Hydrogels made with varying starch-to-monomer ratios, borax levels, and CAN concentrations were labeled as A1–A4, B0–B1, and C1–C4, respectively. Table 1 provides a summary of all sample compositions.

**Table 1:** Hydrogels with Various Compositions

**Samples**	**Starch (g)**	**CAN (g)**	**Borax (g)**	**AM (g)**
A1	5.000	0.700	0.350	15.000
A2	8.000	1.120	0.560	12.000
A3	10.000	1.400	0.700	10.000
A4	12.000	1.680	0.840	8.000
B0	5.000	0.700	0.000	15.000
B1	5.000	0.700	0.250	15.000
B2	5.000	0.700	0.375	15.000
B3	5.000	0.700	0.500	15.000
B4	5.000	0.700	0.625	15.000
C1	5.000	0.300	0.500	15.000
C2	5.000	0.500	0.500	15.000
C3	5.000	0.700	0.500	15.000
C4	5.000	0.900	0.500	15.000

### 2.3 Fourier Transform Infrared Spectroscopy (FTIR)

The FTIR spectra of the starch and hydrogels were measured on a vertex 70 Bruker spectrometer in attenuated total reflectance (ATR) mode. A total of 64 scans were performed for each test with a resolution of 4 cm^–1^ and a spectral range of 4000–400 cm^–1^.

### 2.4 ^1^H NMR Spectroscopy

Proton nuclear magnetic resonance (^1^H NMR, 600 MHz) spectra were obtained using a Bruker Ascend NMR600 spectrometer. Native starch samples were prepared in DMSO-d6, and hydrogel samples in D_2_O. To ensure complete dissolution, the samples were placed in an oven at 65 °C for 2 h prior to measurement.

### 2.5 X-ray Diffractometry (XRD)

The crystalline structure of the samples was analyzed on a Bruker D8 Advanced apparatus. Diffractograms were obtained at a scanning range of 5–50° (2θ) at 40 kV and 40 mA using Cu

Kα radiation (λ = 0.1542 nm).

### 2.6 Scanning Electron Microscopy (SEM)

The hydrogels were freeze-dried and coated with a layer of gold. Their morphologies and microstructures were examined using a Philips XL30 field emission scanning electron microscope equipped with EDAX EDS/EBSD detectors, operating at an accelerating voltage of 10 kV. Nanomeasure software was used to analyze the images and assess the pore size distribution within the hydrogels.

### 2.7 Gel Fraction

The synthesized hydrogels were dried at 60 °C and their initial weight (W_i_) recorded. They were then immersed in distilled water for 3 days until reaching a stable weight, ensuring the removal of soluble components. Afterward, the hydrogels were dried again at 60 °C and weighed (W_d_). The gel fraction (GF) was determined using the following formula (eq 1):
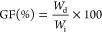
1Here, *W*_i_ represents the initial dry weight of the hydrogel and *W*_d_ is the final dry weight after immersion in water. Each experiment was conducted in triplicate to ensure accuracy.

### 2.8 Swelling Ratio

The swelling ratio of the hydrogels was determined by immersing the initially weighed samples (*W*_i_) in distilled water at room temperature until they reached swelling equilibrium. Afterward, the swollen hydrogels were removed and weighed (*W*_s_). The swelling ratio (SR) was then calculated using the following formula (eq 2):
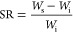
2In this equation, W_i_ represents the hydrogel’s initial dry weight, and W_s_ is the weight of the swollen hydrogel. Each experiment was performed in triplicate for accuracy.

### 2.9 Thermogravimetric Analysis (TGA)

Thermal analysis of the starch and hydrogels was conducted using a TA Instruments (5500) in a nitrogen (N2) atmosphere, covering a temperature range from 30 to 650 °C at a heating rate of 10 °C per minute.

### 2.10 Dynamic Rheological Analysis

The dynamic properties of the hydrogels, including strain sweep, frequency sweep, time sweep, continuous step strain, and temperature sweep, were examined using an Anton Paar MCR302 Rheometer (Anton Paar, Ashland, VA, USA) equipped with a 25 mm parallel plate geometry. Prior to frequency sweep measurements, a dynamic strain sweep from 0.1 to 100% was conducted at an angular frequency (ω) of 10 rad/s, recording the storage modulus (*G*′) to define the linear viscoelastic region, where *G*′ remains unaffected by strain amplitude (γ). A dynamic frequency sweep was performed over an ω range of 0.1–100 rad/s with a strain γ = 1% in this linear viscoelastic region. For self-healing evaluation, dynamic time sweeps were measured at ω = 10 rad/s and γ = 1% from 0 to 600 s before and after cutting the hydrogel into two pieces. Continuous step strain tests were executed in the sequence: γ = 1% (700 s) → γ = 100% (700 s) → γ = 1% (700 s) → γ = 100% (700 s) → γ = 1% (900 s) at ω = 10 rad/s. To assess the thermosensitivity, a temperature sweep was conducted in a heating–cooling–heating cycle (20–95–20–95 °C) at ω = 10 rad/s and γ = 1%, with heating and cooling rates of 5 °C/min. Silicone oil was applied around each sample’s edges to prevent moisture loss.

### 2.11 Statistical Analysis

The means and standard deviations for the gel fraction, swelling ratio, and SEM measurements were calculated. Statistical analysis was conducted using one-way analysis of variance (ANOVA) followed by Duncan’s multiple range test, performed in SPSS software (version 26, IBM, New York, NY, USA). Differences were considered statistically significant at *p* < 0.05.

## 3 Results and Discussion

### 3.1 Hydrogel Morphology

Hydrogels were successfully synthesized using CAN as an initiator to graft acrylamide onto starch in the presence of borax. Borate ions derived from borax created dynamic ester bonds with the hydroxyl groups in starch, linking the starch chains together.

**Figure 1:**
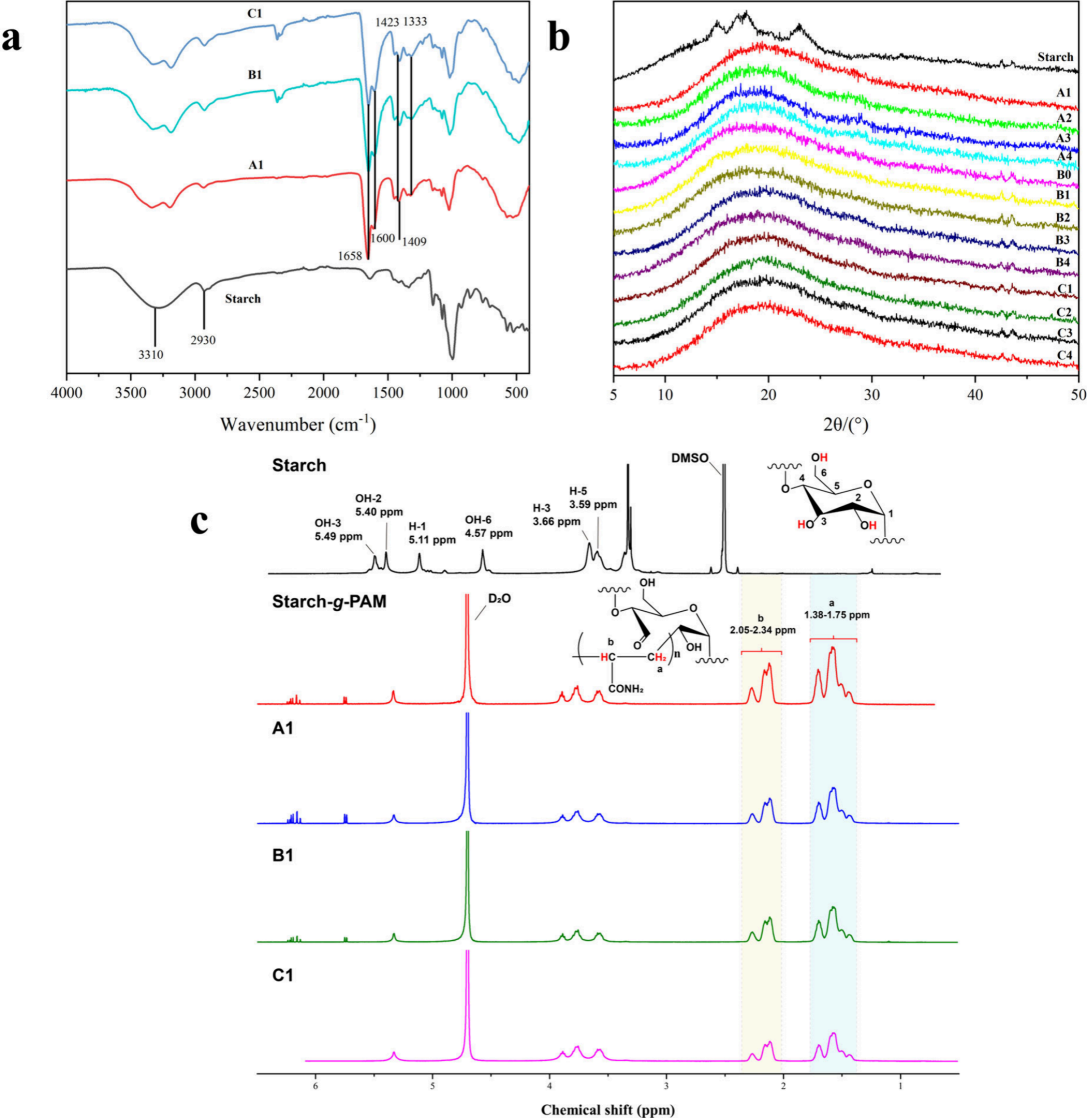
(a) FTIR spectra of native starch and samples A1, B1, and C1; (b) XRD patterns of native starch and samples A1–A4, B0-B4, and C1–C4; and (c) ^1^H NMR spectra of starch, starch-g-PAM, and samples A1, B1, and C1.

Figure 1a presents the FTIR spectra of native starch and the hydrogels. The characteristic absorption peak at 3310 cm^–1^ corresponds to the O–H stretching vibration, the peak at 2930 cm^–1^ is attributed to the C–H stretching vibration, and the peaks at 1625 cm^–1^, 1152 cm^–1^, and 1148 cm^–1^ represent the O–H bending vibration, C–O–C stretching vibration, and glycosidic bond vibration, respectively.^45^ In the spectra of the synthesized hydrogels, new absorption bands appeared at 1658 cm^–1^, 1600 cm^–1^, and 1409 cm^–1^, corresponding to C=O stretching, N–H bending, and C–N stretching, respectively. These bands are indicative of the −CONH_2_ group characteristic of acrylamide.^46^ Additionally, two distinct peaks at 1423 cm^–1^ and 1333 cm^–1^ are associated with the asymmetric stretching of B–O–C bonds.^47^ These findings confirm the successful grafting of acrylamide onto starch and the cross-linking between starch chains and borax.

Figure 1b shows the XRD patterns of native starch and the hydrogels. Native starch displayed typical A-type crystalline structures, with characteristic peaks at 15°, 17°, 18°, and 23°.^48,49^ For all starch hydrogels, the typical crystalline peaks of starch were absent, with only a broad amorphous peak appearing at 20°, reflecting a transition from A-type crystalline to amorphous structures in the X-ray patterns. This alteration suggests a robust interaction between starch chains and borate. The bonding reaction between the hydroxyl groups in starch and borax decreased hydrogen bonding, thus hindering the retrogradation of amylose and/or amylopectin and resulting in reduced starch crystallinity.

Figure 1c illustrates the 1H-NMR spectra for native starch, starch-g-PAM, and hydrogels A1, B1, and C1. The observed chemical shifts at 5.40, 5.49, and 4.57 ppm correspond to the OH-2, OH-3, and OH-6 protons in starch, while the shifts at 5.11, 3.66, and 3.59 ppm relate to the H-1, H-3, and H-5 signals.^50^ Following the reaction, the 1H-NMR spectrum of the starch hydrogels displayed peaks at 1.38–1.75 ppm and 2.05–2.34 ppm, attributed to the −CH_2_ and −CH protons in the PAM structure.^51^ These results confirm the acrylamide was successfully grafted onto the starch molecules.

**Figure 2:**
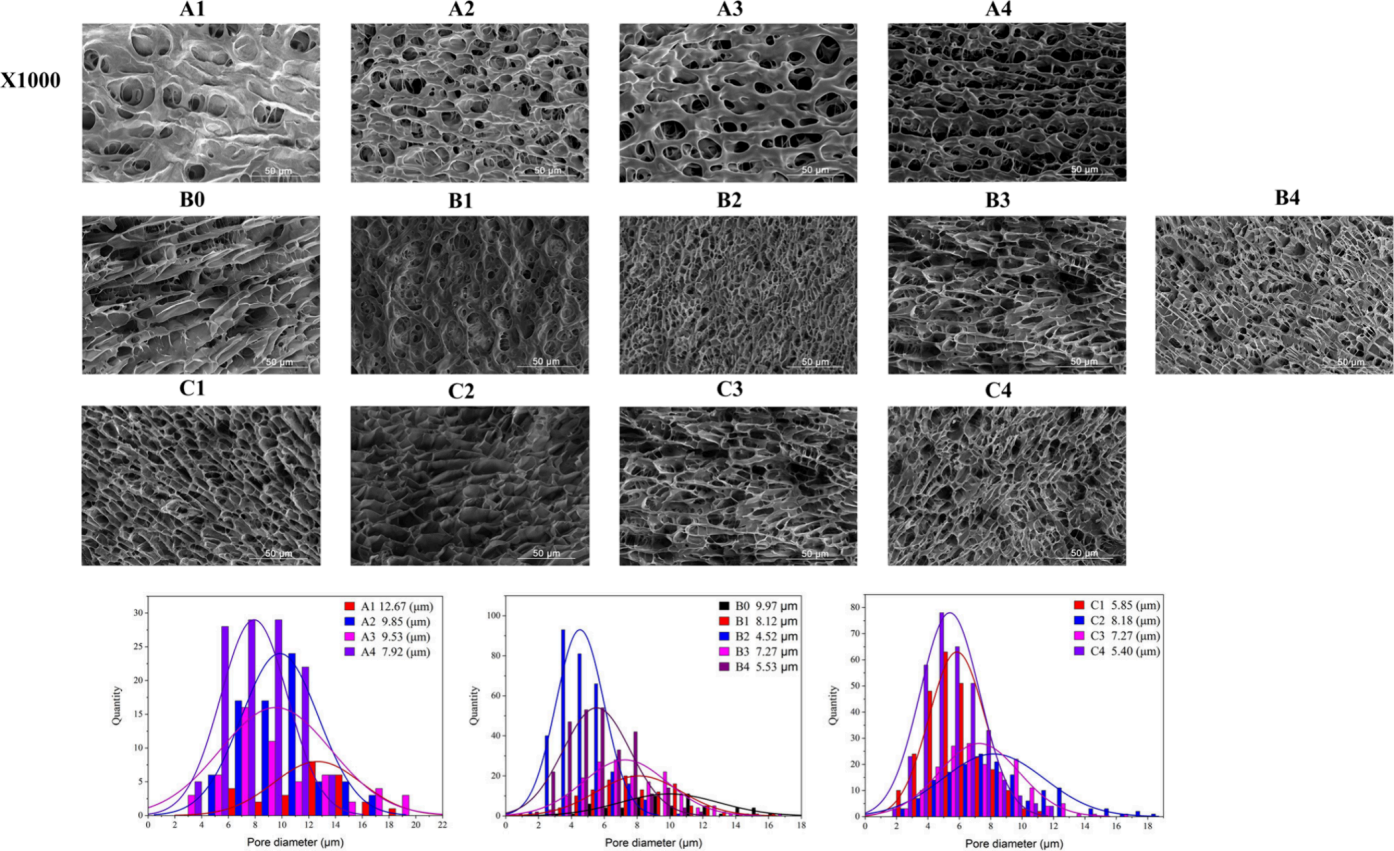
SEM images and pore size distributions of samples A1–A4, B0–B4, and C1–C4.

Figure 2 provides an overview of the internal structures and pore size distributions within the freeze-dried hydrogels. During freeze-drying, the freezable and free water within the hydrogel form ice crystals, which, upon sublimation, create a porous structure in the hydrogel.^52^ The borax cross-linked hydrogels displayed a uniform, interconnected network structure. Table 2 lists the pore diameters for all hydrogels. In samples A1–A4, an increase in the starch-to-monomer ratio led to smaller pore diameters. This effect is likely due to the increased starch content within the hydrogel; the strong hydrophilicity of starch molecules restricted the movement of free water, thereby reducing ice crystal formation and yielding smaller pores after sublimation.^53^ Among samples B0–B4, B2 exhibited the smallest pore diameter (4.52 ± 1.46 μm), which likely contributed to an even distribution of stress within the hydrogel, enhancing its mechanical properties.^54^ The larger pore diameter observed in B3 can be attributed to a higher degree of cross-linking, resulting in a robust network capable of preserving pore structure during ice crystal formation and sublimation. However, an increased borax concentration restricted acrylamide’s access to free radicals, thereby reducing the amount of acrylamide involved in grafting reactions. The predominant component, starch, further limited the mobility of free water molecules, contributing to a reduction in pore diameter. For samples C1–C4, the pore diameter increased and then decreased. This was due to the increased CAN concentration leading to extensive graft polymerization and improved mechanical properties of the hydrogel, which could maintain the pore structure during ice crystal growth and sublimation. A further increase in the CAN concentration increased the cross-linking density, leading to a decrease in the pore diameter.

**Table 2:** Pore Diameter of Hydrogels^a^

**Samples**	**Pore diameter**(μm)	**Samples**	**Pore diameter**(μm)	**Samples**	**Pore diameter**(μm)
		B0	9.97 ± 3.02 ^a^		
A1	12.67 ± 3.28 ^a^	B1	8.12 ± 2.58 ^b^	C1	5.85 ± 1.78 ^c^
A2	9.85 ± 2.89 ^b^	B2	4.52 ± 1.46 ^e^	C2	8.18 ± 3.24 ^a^
A3	9.53 ± 4.15 ^b^	B3	7.27 ± 2.55 ^c^	C3	7.27 ± 2.55 ^b^
A4	7.92 ± 2.51 ^c^	B4	5.53 ± 2.04 ^d^	C4	5.40 ± 1.90 ^d^

a Values in the same column with different letters are significantly different (*p* < 0.05).

### 3.2 Gel Fraction and Swelling Ratio

**Figure 3:**
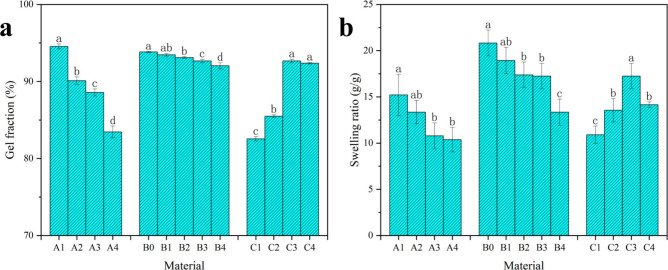
(a) Gel fractions of samples A1–A4, B0–B4, and C1–C4. (b) Swelling ratio of samples A1–A4, B0–B4, and C1–C4. Values with different letters are significantly different (*p* < 0.05).

The influence of the starch-to-monomer ratio, borax concentration, and CAN concentration on the gel fraction percentage is shown in Figure 3a. Increasing the starch-to-monomer ratio resulted in a lower gel fraction. This was due to the decreased amount of acrylamide involved in the grafting reactions, which led to shorter polyacrylamide (PAM) chains being grafted onto the starch. In addition, the starch used in this research mainly consisted of highly branched amylopectin. Therefore, less entanglement of the polymer chains reduced the strength of the hydrogel.^55^ Likewise, the gel fraction decreased with higher borax concentrations. It appears that borate ions quickly formed borate ester bonds with the hydroxyl groups of starch, creating a high-viscosity starch-borax complex. This complex likely trapped free radicals within “cages,″ restricting acrylamide’s access to these radicals for grafting onto the starch chains, resulting in a weaker hydrogel network.^56^ In addition, all these differences are only slight since the increased borax concentration enhanced the cross-link density. Moreover, the gel fraction increased with increasing CAN concentration. This could be attributed to an increased number of free radicals on the starch chains, which facilitated graft polymerization. Figure 3b shows the relationships between the swelling ratio and the starch-to-monomer ratio, borax concentration, and CAN concentration. The swelling ratio decreased as the starch-to-monomer ratio increased. This was due to the highly branched amylopectin hindering water movement in the hydrogel and the shorter grafted hydrophilic PAM chains, which led to a lower swelling ratio. The swelling ratio declined as borax concentration increased, likely because higher borax levels raised the cross-link density and reduced the spacing between polymer chains. This limited the hydrogel network’s ability to expand, leading to a lower swelling ratio. In addition, the swelling ratio increased with increasing CAN concentration and then decreased. The initial increase in the swelling ratio might be attributed to the increased number of free radicals on the starch chains, leading to extensive graft polymerization.^57^ A further increase in the CAN concentration resulted in a faster reaction rate and led to a terminating step reaction between the starch chains, which increased the cross-link density and decreased the swelling ratio.

### 3.3 Thermal Stability

**Figure 4:**
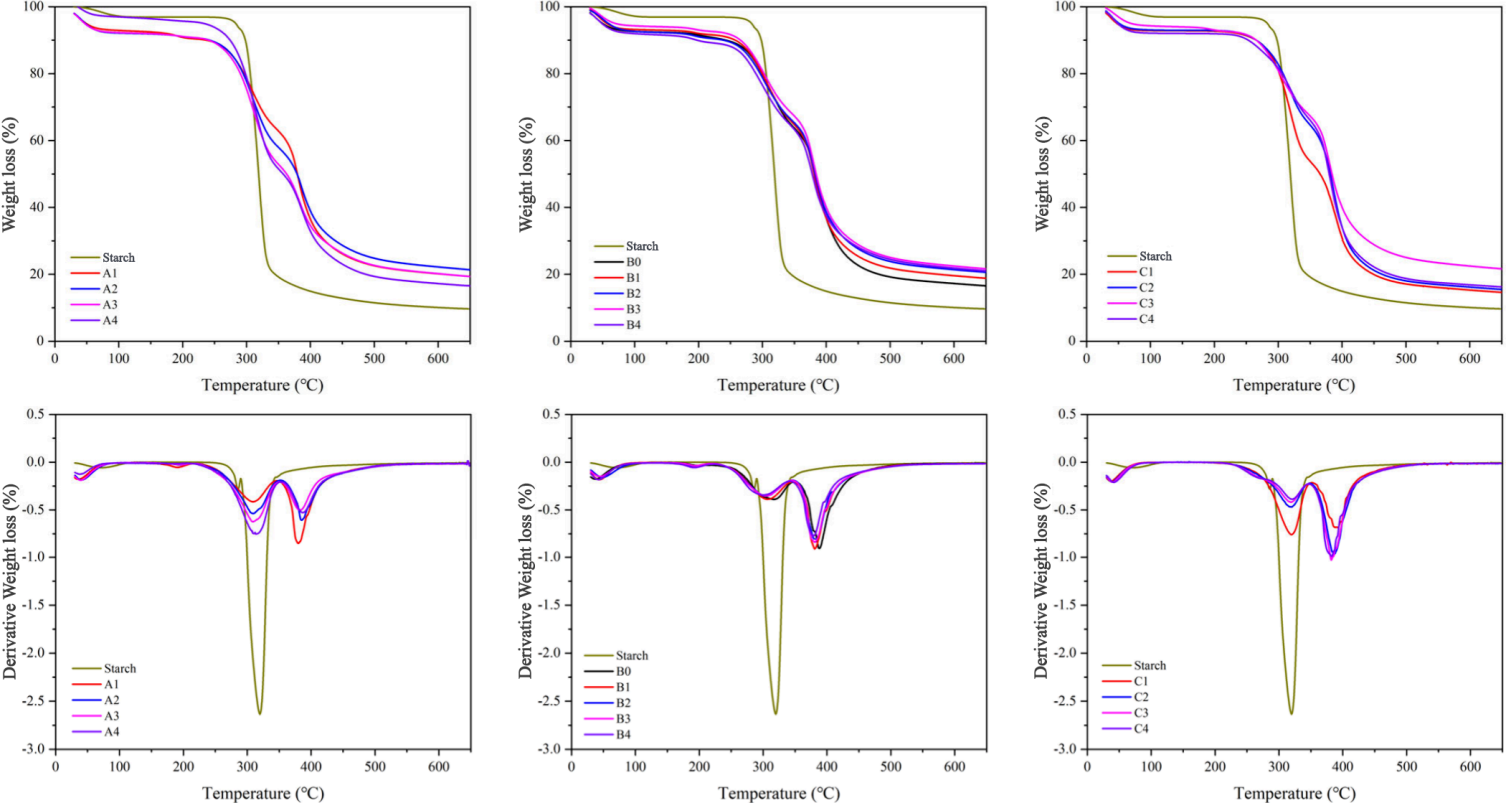
TGA and DTG curves of native starch and samples A1–A4, B0–B4, and C1–C4.

Figure 4 presents the TGA and derivative thermogravimetry (DTG) curves for native starch and samples A1–A4, B0–B4, and C1–C4. For native starch, the TGA curve shows two primary stages of decomposition: the first stage, ranging from 25 to 250 °C, is linked to water loss, while the second stage, from 250 to 350 °C, corresponds to the thermal degradation of the starch structure.^58^ In the starch hydrogels, three distinct stages of degradation were observed: the first stage (25–250 °C) involved water molecule evaporation, the second stage (250–350 °C) was associated with the breakdown of the starch matrix, and the third stage (350–550 °C) was linked to the degradation of the PAM grafted onto the starch chains.^59^ The temperature at which peak decomposition occurred for starch within the hydrogels was lower than that for native starch, likely because the crystalline structure of starch granules was disrupted during gelatinization. Furthermore, native starch showed a weight loss of 90%, whereas the borax cross-linked hydrogels retained a greater portion of their weight after heating, suggesting improved thermal stability as a result of borax integration.The percentage add-on (AO) and grafting ratio (GR) of the hydrogels, calculated based on the percentage weight loss observed in the TGA analysis, are provided in Table 3.^60^ In the analysis of starch hydrogels, the weight loss percentage at stage 2 was noted as W_s2_, and at stage 3 as W_s3_. The percentage add-on (AO) and grafting ratio (GR) were determined using the following formulas (eqs 3 and 4):
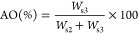
3
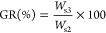
4

**Table 3:** Percentage Add On (AO) and Grafting Ratio (GR) of Hydrogels

**Samples**	**AO (%)**	**GR (%)**	**Samples**	**AO (%)**	**GR (%)**	**Samples**	**AO (%)**	**GR (%)**
			B0	63.4	173.1			
A1	62.4	165.9	B1	64.5	181.3	C1	48.8	95.3
A2	52.9	112.1	B2	65.7	191.1	C2	63.7	175.7
A3	47.3	89.9	B3	66.4	197.4	C3	66.4	197.4
A4	43.3	76.5	B4	65.1	186.2	C4	66.7	200.1

The AO and GR decreased as the starch-to-monomer ratio increased, which could be attributed to the decreased amount of acrylamide involved in the grafting reactions. The AO and GR increased with increasing borax concentration and then decreased. One possible explanation is that, upon adding borax to the solution, borate ions quickly formed ester bonds with the hydroxyl groups in starch, reducing the spacing between starch chains. This restricted the movement of free radicals between the starch chains, increasing the medium’s viscosity. In such a high-viscosity environment, free radicals can become trapped in “cages”, blocking acrylamide’s access to these radicals.^56^ Since there were many free radicals on the starch chains, when the borax concentration increased, the homopolymerization of acrylamide decreased, and the AO and GR increased. However, increasing the borax concentration beyond a certain point reduced the involvement of acrylamide in the grafting reactions, leading to lower AO and GR values. Moreover, increasing the CAN concentration resulted in increased AO and GR. At a lower concentration of CAN, there were only a few free radicals on the starch chains, and not enough acrylamide participated in the reaction. A further increase in CAN produced more free radicals, which facilitated graft polymerization and increased the AO and GR content.

### 3.4 Rheological Properties

**Figure 5:**
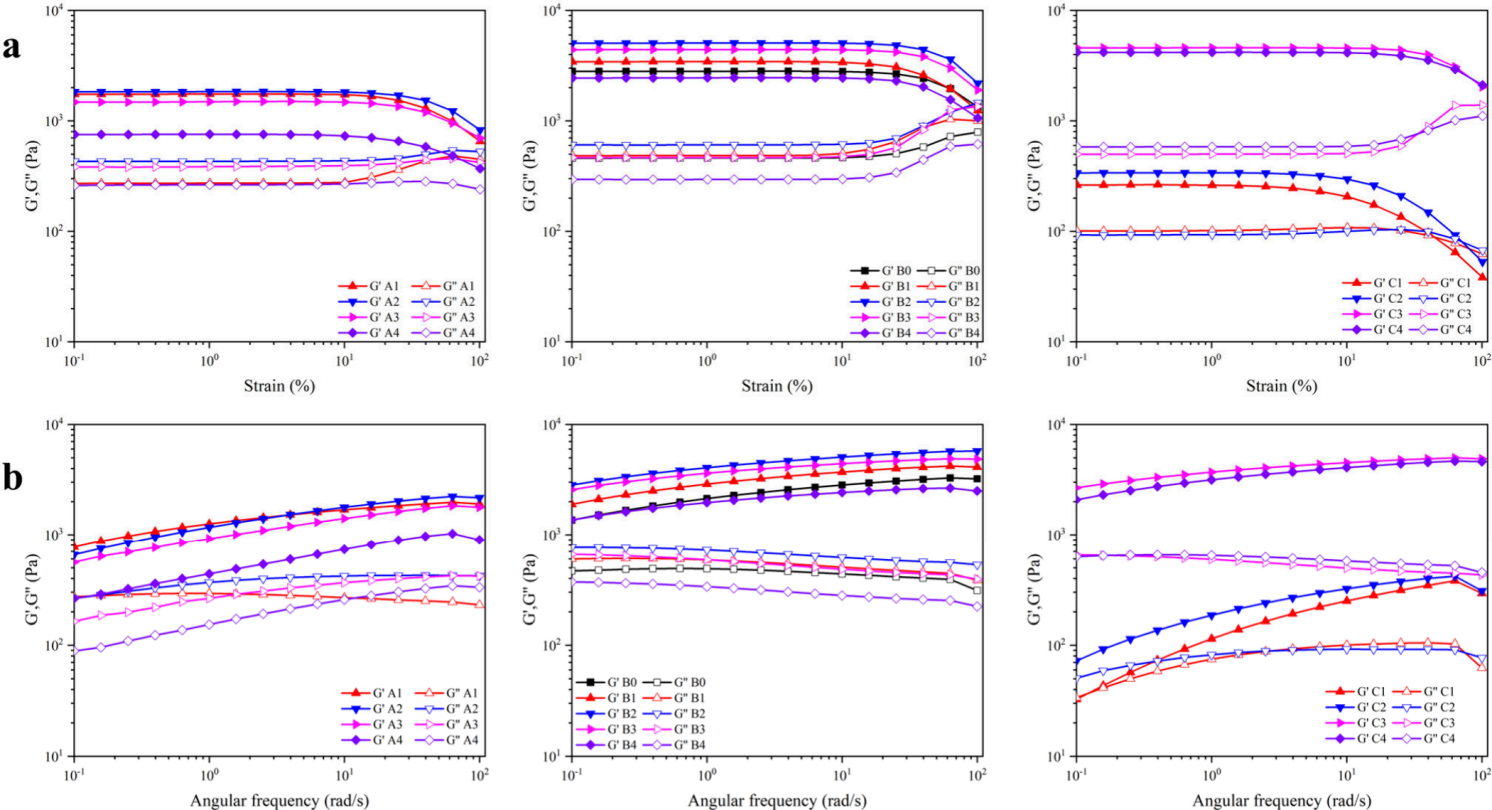
(a) Dynamic strain sweep curves at ω = 10 rad/s. (b) Dynamic frequency sweep curves at γ = 1%.

The study examined how variations in starch-to-monomer ratio, borax concentration, and CAN concentration affect the rheological characteristics of starch hydrogels. To determine the linear viscoelastic range, dynamic strain sweep tests were conducted, as illustrated in Figure 5a, which shows the storage modulus (*G*′) and loss modulus (*G*″) across strain amplitudes from 0.1% to 100% at a fixed frequency of 10 rad/s. Up to a strain of 1%, *G*′ values were stable for all samples, so this strain level was applied in dynamic frequency sweep tests to ensure hydrogel deformation stayed within the linear viscoelastic range.^61^ Consistently, *G*′ exceeded *G*″, demonstrating the hydrogels’ predominantly elastic properties. Initially, both *G*′ and *G*″ rose with an increasing starch-to-monomer ratio, then declined afterward.

The initial improvement could be attributed to the increased entanglement of starch chains, which enhanced the toughness of the hydrogels. For samples A3 and A4, the decreased amount of acrylamide involved in the grafting reactions reduced the strength of the hydrogel, which led to a decrease in *G*′ and *G*″. Similarly, *G*′ and *G*″ first increased and then decreased with increasing borax concentration. Compared with the other hydrogels, B2 demonstrated the highest Gmax′ value (∼5072 Pa), which was nearly 1.8-fold greater than that of the B0 hydrogel (∼2816 Pa), suggesting the significant reinforcing effect of borax. The *G*′ and *G*″ of sample B4 were even lower than those of sample B0. This can be attributed to the tendency of higher borax concentrations to trap free radicals within “cages,″ which restricts acrylamide’s access to these radicals. Consequently, the reduced availability of acrylamide for grafting reactions weakens the hydrogel structure. For samples C1–C4, the addition of CAN pronouncedly increased the *G*′ and *G*″ of the hydrogel; C3 demonstrated the highest Gmax′ value (∼4596 Pa), and this value was almost 17.6 times greater than that of the C1 hydrogel (∼261 Pa). The difference relates to the lack of sufficient free radicals in the polymerization reactions at a low concentration of CAN, and the resulting three-dimensional network that cannot be formed efficiently. A higher CAN concentration facilitated graft polymerization, leading to a vast increase in *G*′ and *G*″.

Figure 5b illustrates the storage modulus (*G*′) and loss modulus (*G*″) as functions of angular frequency (ω = 0.1–100 rad/s) at a 1% strain. Across the entire ω range, *G*′ values for all hydrogels exceeded *G*″ values, indicating their elastic nature. Unlike permanently cross-linked hydrogels, which show frequency-independent behavior, these hydrogels displayed frequency-dependent moduli, suggesting dynamic cross-linking.^62^ As ω increased, the *G*′ of most hydrogels continued to rise until reaching a plateau, characteristic of polymer entanglements.^63^ However, for samples A4, B4, C1, and C2, the plateau was not observed, likely due to their lower network strength, which hindered recovery at higher ω values. Additionally, the trends in *G*′ and *G*″ across frequency were consistent with those seen in the strain sweep tests. These findings confirm the presence of polymer chain entanglements and reversible borate ester bonds in the hydrogels.

### 3.5 Self-Healing Properties

**Figure 6:**
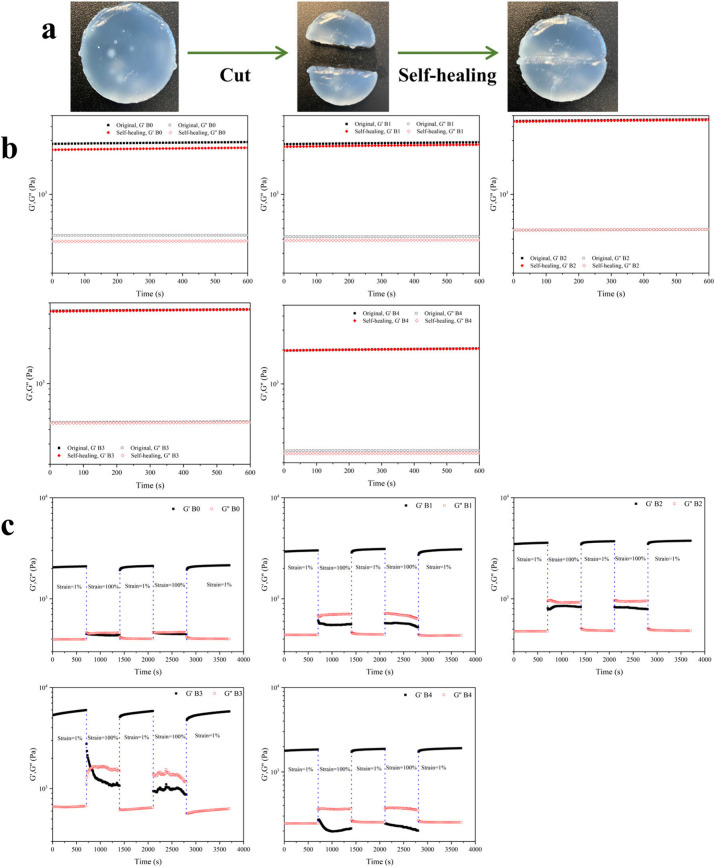
(a) Self-healing pictures of B3 hydrogel. (b) *G*′ and *G*″ versus time for the original and self-healing B0–B4 hydrogels. (c) Continuous step-strain measurements of the B0–B4 hydrogel at strains of 1% and 100%.

The hydrogels’ self-healing ability was evaluated using rheological tests, selecting samples B0–B4 as representatives, as shown in Figure 6. In Figure 6a, the B3 hydrogel was sliced into two parts and rejoined at room temperature, with the halves successfully reconnecting without any external intervention. Figure 6b illustrates the *G*′ and *G*″ values over time for both the original and self-healed B0–B4 hydrogels. The *G*′ and *G*″ values for self-healed B1–B4 hydrogels closely matched those of the original samples, indicating successful structural recovery. Conversely, the B0 hydrogel, which did not contain borax, was only able to partially restore its mechanical properties, relying solely on hydrogen bonds within its network. As illustrated in Figure 6c, all hydrogels showed self-healing recovery when subjected to strain-induced damage. At low strain (γ = 1%), the B3 hydrogel exhibited a solid structure, with *G*′ around 5500 Pa and *G*″ around 650 Pa. Under higher strain (γ = 100%), *G*′ decreased from 5500 Pa to approximately 1000 Pa, indicating a reduction in mechanical stability. Upon returning to low strain (γ = 1%), both *G*′ and *G*″ immediately recovered to their initial values, highlighting the hydrogel’s self-recovery ability. These results highlight the hydrogel’s self-healing capability, which is due to the presence of hydrogen bonds and reversible borate ester bonds within its structure.

### 3.6 Thermosensitivity Property

**Figure 7:**
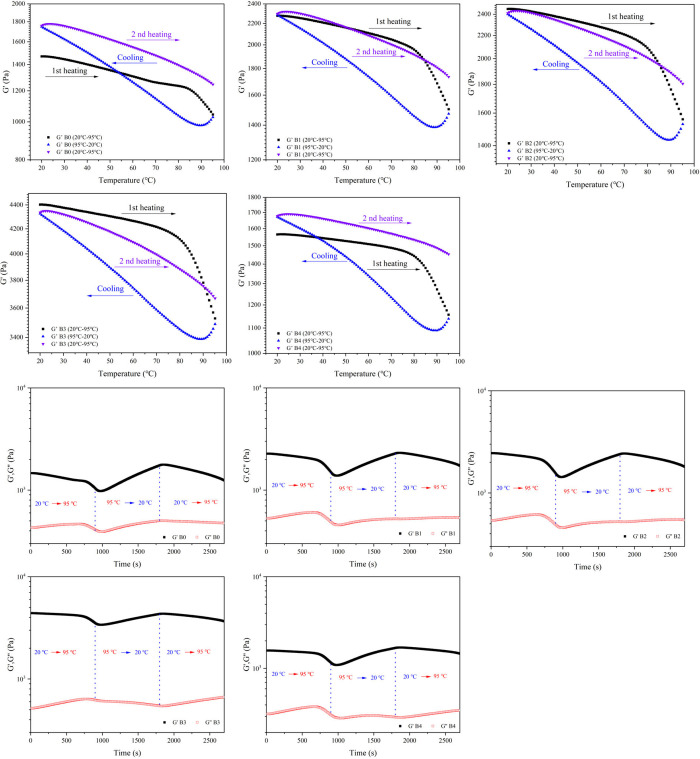
Temperature dependence of *G*′ and *G*″ for the B0–B4 hydrogel during a heating–cooling–heating circle at ω = 10 rad/s and γ = 1%.

**Figure 8:**
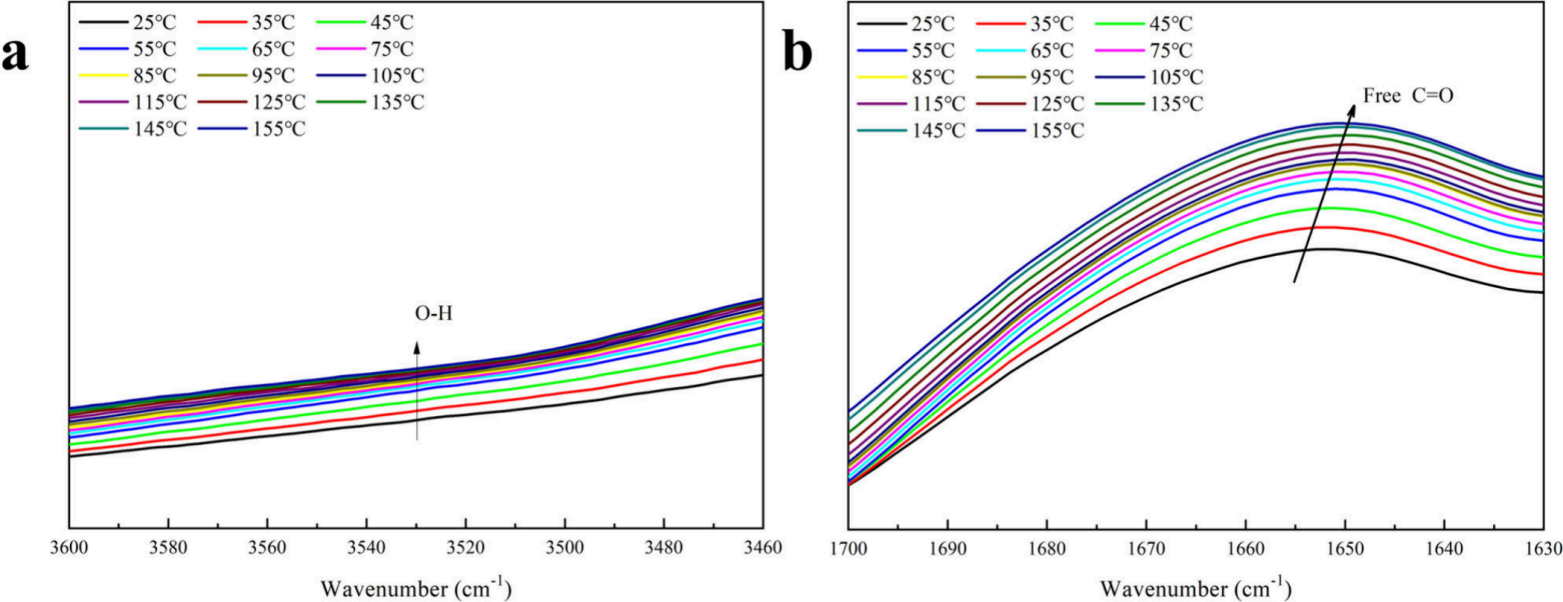
Variable temperature FTIR spectra of B3 hydrogels in the (a) 3600–3460 cm^–1^ region and (b) 1700–1630 cm^–1^ region.

As depicted in Figure 7, the B0–B4 hydrogel was used as an example to illustrate the thermosensitivity of the hydrogel. Throughout the heating–cooling–heating cycle, *G*′ consistently exceeded *G*″, indicating the hydrogel’s elastic-like behavior. During the initial heating phase, as the temperature rose from 20 to 95 °C, *G*′ showed a marked decrease with increasing temperature. In the subsequent cooling phase, *G*′ nearly returned to its initial value, suggesting that the hydrogel could regain its mechanical properties. Furthermore, after the heating–cooling–heating sequence, the *G*_max_′ values of B0 and B4 increased noticeably, likely due to internal water evaporation, as these hydrogels exhibited relatively weak mechanical properties. Figure 8 presents the variable temperature FTIR spectra of the B3 hydrogel, where the peak at 3500 cm^–1^, associated with O–H stretching, intensified with rising temperature. This increase is likely due to the dissociation of hydrogen and borate ester bonds, leading to a higher concentration of hydroxyl groups. Additionally, the peak at 1658 cm^–1^, corresponding to free carbonyl groups, grew as the temperature increased from 25 to 155 °C, indicating the weakening and dissociation of hydrogen bonds.^64^ These findings reveal the hydrogel’s thermal responsiveness, attributable to hydrogen bonding and reversible, exothermic interactions between starch hydroxyl groups and borate ions.

## 4 Conclusions

In summary, a novel acrylamide-grafted starch hydrogel was successfully developed using borax as a cross-linker. The dynamic borate ester bonds formed between starch hydroxyl groups and borax, along with hydrogen bonding, imparted self-healing and thermal responsiveness to the hydrogel. The influence of starch-to-monomer ratio, borax concentration, and CAN concentration on the hydrogel’s mechanical properties was analyzed through rheological testing. Results indicated that borax significantly enhanced hydrogel strength. Notably, moderate borax levels increased cross-link density and reduced acrylamide homopolymerization. Higher borax concentrations, however, trapped free radicals within “cages”, restricting acrylamide’s access to free radicals, reducing grafting, and yielding a weaker hydrogel. Increased CAN concentration promoted graft polymerization, as seen in the *G*′ of the C3 hydrogel, which was nearly 17.6 times that of the C1 hydrogel. Additionally, the B3 hydrogel demonstrated recovery after 100% shear strain and showed thermal responsiveness due to reversible, exothermic interactions between starch hydroxyl groups and borate ions. This work contributes to advancing closed-loop material cycles and expands the potential applications of starch-based hydrogels in agriculture, sensor technology, and wastewater treatment.

## References

[ref1] Zhou Y. M.; Fu S. Y.; Zhang L. L.; Zhan H. Y. Superabsorbent nanocomposite hydrogels made of carboxylated cellulose nanofibrils and CMC-g-p(AA-co-AM). Carbohydr. Polym. 2013, 97 (2), 429–435. 10.1016/j.carbpol.2013.04.088. 23911467

[ref2] Bao Y.; Ma J. Z.; Li N. Synthesis and swelling behaviors of sodium carboxymethyl cellulose-g-poly(AA-co-AM-co-AMPS)/MMT superabsorbent hydrogel. Carbohydr. Polym. 2011, 84 (1), 76–82. 10.1016/j.carbpol.2010.10.061.

[ref3] Dai H. J.; Huang H. H. Modified pineapple peel cellulose hydrogels embedded with sepia ink for effective removal of methylene blue. Carbohydr. Polym. 2016, 148, 1–10. 10.1016/j.carbpol.2016.04.040. 27185109

[ref4] Guilherme M. R.; Aouada F. A.; Fajardo A. R.; Martins A. F.; Paulino A. T.; Davi M. F. T.; Rubira A. F.; Muniz E. C. Superabsorbent hydrogels based on polysaccharides for application in agriculture as soil conditioner and nutrient carrier: A review. Eur. Polym. J. 2015, 72, 365–385. 10.1016/j.eurpolymj.2015.04.017.

[ref5] Zhang Z.; Abidi N.; Lucia L.; Chabi S.; Denny C. T.; Parajuli P.; Rumi S. S. Cellulose/nanocellulose superabsorbent hydrogels as a sustainable platform for materials applications: A mini-review and perspective. Carbohydr. Polym. 2023, 299, 120140 10.1016/j.carbpol.2022.120140. 36876763

[ref6] Xu T.; Liu K.; Sheng N.; Zhang M. H.; Liu W.; Liu H. Y.; Dai L.; Zhang X. Y.; Si C. L.; Du H. S.; et al. Biopolymer-based hydrogel electrolytes for advanced energy storage/conversion devices: Properties, applications, and perspectives. Energy Storage Mater. 2022, 48, 244–262. 10.1016/j.ensm.2022.03.013.

[ref7] Sawut A.; Yimit M.; Sun W. F.; Nurulla I. Photopolymerisation and characterization of maleylatedcellulose-g-poly(acrylic acid) superabsorbent polymer. Carbohydr. Polym. 2014, 101, 231–239. 10.1016/j.carbpol.2013.09.054. 24299769

[ref8] Boetje L.; Lan X. H.; van Dijken J.; Kaastra G.; Polhuis M.; Loos K. Thiol-Ene Click Cross-linking of Starch Oleate Films for Enhanced Properties. Biomacromolecules 2023, 24 (12), 5578–5588. 10.1021/acs.biomac.3c00507. 37934174 PMC10716852

[ref9] Zhang Y. N.; Cui J. Y.; Xu S. A. Effects of chain structures of corn starches on starch-based superabsorbent polymers. Starch/Stärke 2015, 67 (11–12), 949–957. 10.1002/star.201500088.

[ref10] Boetje L.; Lan X. H.; van Dijken J.; Woortman A. J. J.; Popken T.; Polhuis M.; Loos K. Starch ester film properties: The role of the casting temperature and starch its molecular weight and amylose content. Carbohydr. Polym. 2023, 316, 121043 10.1016/j.carbpol.2023.121043. 37321736

[ref11] Boetje L.; Lan X. H.; van Dijken J.; Polhuis M.; Loos K. Synthesis and Properties of Fully Biobased Crosslinked Starch Oleate Films. Polymers 2023, 15 (11), 2467 10.3390/polym15112467. 37299266 PMC10255509

[ref12] Nasution H.; Harahap H.; Dalimunthe N. F.; Ginting M. H. S.; Jaafar M.; Tan O. O. H.; Aruan H. K.; Herfananda A. L. Hydrogel and Effects of Crosslinking Agent on Cellulose-Based Hydrogels: A Review. Gels 2022, 8 (9), 568 10.3390/gels8090568. 36135281 PMC9498307

[ref13] Lan X. H.; Li W. J.; Ye C. N.; Boetje L.; Pelras T.; Silvianti F.; Chen Q.; Pei Y. T.; Loos K. Scalable and Degradable Dextrin-Based Elastomers for Wearable Touch Sensing. ACS Appl. Mater. Interfaces 2023, 15 (3), 4398–4407. 10.1021/acsami.2c15634. 36514844 PMC9880951

[ref14] Boetje L.; Lan X. H.; Silvianti F.; van Dijken J.; Polhuis M.; Loos K. A more efficient synthesis and properties of saturated and unsaturated starch esters. Carbohydr. Polym. 2022, 292, 119649 10.1016/j.carbpol.2022.119649. 35725159

[ref15] Ahmadi-Abhari S.; Woortman A. J. J.; Hamer R. J.; Oudhuis A.; Loos K. Influence of lysophosphatidylcholine on the gelation of diluted wheat starch suspensions. Carbohydr. Polym. 2013, 93 (1), 224–231. 10.1016/j.carbpol.2012.05.020. 23465923

[ref16] Lu K.; Zhu J.; Bao X. Y.; Liu H. S.; Yu L.; Chen L. Effect of starch microstructure on microwave-assisted esterification. Int. J. Biol. Macromol. 2020, 164, 2550–2557. 10.1016/j.ijbiomac.2020.08.099. 32798547

[ref17] Yassaroh Y.; Woortman A. J. J.; Loos K. Physicochemical properties of heat-moisture treated, stearic acid complexed starch: The effect of complexation time and temperature. Int. J. Biol. Macromol. 2021, 175, 98–107. 10.1016/j.ijbiomac.2021.01.124. 33508365

[ref18] Anisa S.; Woortman A. J. J.; Loos K.; Rachmawati R. Investigation of Physicochemical Properties of Tapioca Starch-Methyl Myristate Complexes. Starch/Stärke 2023, 75 (11–12), 11 10.1002/star.202300043.

[ref19] Chen X. Y.; Ji N.; Li F.; Qin Y.; Wang Y. F.; Xiong L.; Sun Q. J. Dual Cross-Linked Starch-Borax Double Network Hydrogels with Tough and Self-Healing Properties. Foods 2022, 11 (9), 1315 10.3390/foods11091315. 35564038 PMC9103891

[ref20] Yassaroh Y.; Nurhaini F. F.; Woortman A. J. J.; Loos K. Physicochemical properties of heat-moisture treated, sodium stearate complexed starch: The effect of sodium stearate concentration. Carbohydr. Polym. 2021, 269, 118263 10.1016/j.carbpol.2021.118263. 34294296

[ref21] Ahmadi-Abhari S.; Woortman A. J. J.; Hamer R. J.; Loos K. Assessment of the influence of amylose-LPC complexation on the extent of wheat starch digestibility by size-exclusion chromatography. Food Chem. 2013, 141 (4), 4318–4323. 10.1016/j.foodchem.2013.06.088. 23993621

[ref22] Zhao L.; Ren Z. J.; Liu X.; Ling Q. J.; Li Z. J.; Gu H. B. A Multifunctional, Self-Healing, Self-Adhesive, and Conductive Sodium Alginate/Poly(vinyl alcohol) Composite Hydrogel as a Flexible Strain Sensor. ACS Appl. Mater. Interfaces 2021, 13 (9), 11344–11355. 10.1021/acsami.1c01343. 33620195

[ref23] Li N.; Liu C. J.; Chen W. Facile Access to Guar Gum Based Supramolecular Hydrogels with Rapid Self-Healing Ability and Multistimuli Responsive Gel-Sol Transitions. J. Agric. Food Chem. 2019, 67 (2), 746–752. 10.1021/acs.jafc.8b05130. 30571099

[ref24] Ye C. N.; Yan F.; Lan X. H.; Rudolf P.; Voet V. S. D.; Folkersma R.; Loos K. Novel MXene sensors based on fast healing vitrimers. Appl. Mater. Today 2022, 29, 101683 10.1016/j.apmt.2022.101683.

[ref25] Ai J. Y.; Li K.; Li J. B.; Yu F.; Ma J. Super flexible, fatigue resistant, self-healing PVA/xylan/borax hydrogel with dual-crosslinked network. Int. J. Biol. Macromol. 2021, 172, 66–73. 10.1016/j.ijbiomac.2021.01.038. 33434549

[ref26] Li M. Y.; Han X.; Fan Z. W.; Zhang Y.; Li Q. L.; Xie G. X. Autonomous ultrafast-self-healing hydrogel for application in multiple environments. Colloids Surf. A Physicochem. Eng. Asp. 2021, 631, 127669 10.1016/j.colsurfa.2021.127669.

[ref27] Long T. J.; Li Y. X.; Fang X.; Sun J. Q. Salt-Mediated Polyampholyte Hydrogels with High Mechanical Strength, Excellent Self-Healing Property, and Satisfactory Electrical Conductivity. Adv. Funct. Mater. 2018, 28 (44), 9 10.1002/adfm.201804416.

[ref28] Nakahata M.; Takashima Y.; Yamaguchi H.; Harada A. Redox-responsive self-healing materials formed from host-guest polymers. Nat. Commun. 2011, 2, 6 10.1038/ncomms1521. PMC3207205 22027591

[ref29] Chao A.; Negulescu J.; Zhang D. H. Dynamic Covalent Polymer Networks Based on Degenerative Imine Bond Exchange: Tuning the Malleability and Self-Healing Properties by Solvent. Macromolecules 2016, 49 (17), 6277–6284. 10.1021/acs.macromol.6b01443.

[ref30] Chen Y. L.; Guan Z. B. Multivalent hydrogen bonding block copolymers self-assemble into strong and tough self-healing materials. Chem. Commun. 2014, 50 (74), 10868–10870. 10.1039/C4CC03168G. 25090104

[ref31] Huang S. Q.; Su S. Y.; Gan H. B.; Wu L. J.; Lin C. H.; Xu D. Y.; Zhou H. F.; Lin X. L.; Qin Y. L. Facile fabrication and characterization of highly stretchable lignin-based hydroxyethyl cellulose self-healing hydrogel. Carbohydr. Polym. 2019, 223, 115080 10.1016/j.carbpol.2019.115080. 31427024

[ref32] Shao C. Y.; Wang M.; Chang H. L.; Xu F.; Yang J. A Self-Healing Cellulose Nanocrystal-Poly(ethylene glycol) Nanocomposite Hydrogel via Diels-Alder Click Reaction. ACS Sustain Chem. Eng. 2017, 5 (7), 6167–6174. 10.1021/acssuschemeng.7b01060.

[ref33] Pepels M.; Filot I.; Klumperman B.; Goossens H. Self-healing systems based on disulfide-thiol exchange reactions. Polym. Chem. 2013, 4 (18), 4955–4965. 10.1039/c3py00087g.

[ref34] Cheng H. L.; Fan Z.; Wang Z. Y.; Guo Z. J.; Jiang J. A.; Xie Y. M. Highly stretchable, fast self-healing nanocellulose hydrogel combining borate ester bonds and acylhydrazone bonds. Int. J. Biol. Macromol. 2023, 245, 125471 10.1016/j.ijbiomac.2023.125471. 37336381

[ref35] Rumon M. M. H.; Akib A. A.; Sultana F.; Moniruzzaman M.; Niloy M. S.; Shakil M. S.; Roy C. K. Self-Healing Hydrogels: Development, Biomedical Applications, and Challenges. Polymers 2022, 14 (21), 4539 10.3390/polym14214539. 36365532 PMC9654449

[ref36] Ren Z. J.; Ke T.; Ling Q. J.; Zhao L.; Gu H. B. Rapid self-healing and self-adhesive chitosan-based hydrogels by host-guest interaction and dynamic covalent bond as flexible sensor. Carbohydr. Polym. 2021, 273, 118533 10.1016/j.carbpol.2021.118533. 34560946

[ref37] Lu B. L.; Lin F. C.; Jiang X.; Cheng J. J.; Lu Q. L.; Song J. B.; Chen C.; Huang B. One-Pot Assembly of Microfibrillated Cellulose Reinforced PVA-Borax Hydrogels with Self-Healing and pH-Responsive Properties. ACS Sustain Chem. Eng. 2017, 5 (1), 948–956. 10.1021/acssuschemeng.6b02279.

[ref38] Seidi F.; Jin Y. C.; Han J. Q.; Saeb M. R.; Akbari A.; Hosseini S. H.; Shabanian M.; Xiao H. N. Self-healing Polyol/Borax Hydrogels: Fabrications, Properties and Applications. Chem. Rec. 2020, 20 (10), 1142–1162. 10.1002/tcr.202000060.

[ref39] Mate C. J.; Mishra S. Synthesis of borax cross-linked Jhingan gum hydrogel for remediation of Remazol Brilliant Blue R (RBBR) dye from water: Adsorption isotherm, kinetic, thermodynamic and biodegradation studies. Int. J. Biol. Macromol. 2020, 151, 677–690. 10.1016/j.ijbiomac.2020.02.192. 32084480

[ref40] Dai L.; Nadeau B.; An X. Y.; Cheng D.; Long Z.; Ni Y. H. Silver nanoparticles-containing dual-function hydrogels based on a guar gum-sodium borohydride system. Sci. Rep 2016, 6, 6 10.1038/srep36497. 27819289 PMC5098157

[ref41] Koga K.; Takada A.; Nemoto N. Dynamic light scattering and dynamic viscoelasticity of poly(vinyl alcohol) in aqueous borax solutions. 5. Temperature effects. Macromolecules 1999, 32 (26), 8872–8879. 10.1021/ma990493w.

[ref42] Qin Y.; Wang J. P.; Qiu C.; Xu X. M.; Jin Z. Y. A Dual Cross-Linked Strategy to Construct Moldable Hydrogels with High Stretchability, Good Self-Recovery, and Self-Healing Capability. J. Agric. Food Chem. 2019, 67 (14), 3966–3980. 10.1021/acs.jafc.8b05147. 30888158

[ref43] Tantiwatcharothai S.; Prachayawarakorn J. Property improvement of antibacterial wound dressing from basil seed (O. basilicum L.) mucilage- ZnO nanocomposite by borax crosslinking. Carbohydr. Polym. 2020, 227, 115360 10.1016/j.carbpol.2019.115360. 31590866

[ref44] Wu J. Z.; Liu Y. J.; Hua S. M.; Meng F. J.; Ma Q. L.; Song S. L.; Che Y. J. Dynamic Cross-Linking Network Construction of Carboxymethyl Starch Enabling Temperature and Strain Bimodal Film Sensors. ACS Appl. Mater. Interfaces 2023, 15 (13), 17293–17300. 10.1021/acsami.3c01918. 36951487

[ref45] Kumar K.; Loos K. Deciphering Structures of Inclusion Complexes of Amylose with Natural Phenolic Amphiphiles. ACS Omega 2019, 4 (18), 17807–17813. 10.1021/acsomega.9b02388. 31681887 PMC6822131

[ref46] Bao X. Y.; Yu L.; Shen S. R. L.; Simon G. P.; Liu H. S.; Chen L. How rheological behaviors of concentrated starch affect graft copolymerization of acrylamide and resultant hydrogel. Carbohydr. Polym. 2019, 219, 395–404. 10.1016/j.carbpol.2019.05.034. 31151540

[ref47] Spoljaric S.; Salminen A.; Luong N. D.; Seppälä J. Stable, self-healing hydrogels from nanofibrillated cellulose, poly(vinyl alcohol) and borax via reversible crosslinking. Eur. Polym. J. 2014, 56, 105–117. 10.1016/j.eurpolymj.2014.03.009.

[ref48] Wang S. Q.; Wu T. H.; Cui W. J.; Liu M. H.; Wu Y. Z.; Zhao C. B.; Zheng M. Z.; Xu X. Y.; Liu J. S. Structure and in vitro digestibility on complex of corn starch with soy isoflavone. Food Sci. Nutr. 2020, 8 (11), 6061–6068. 10.1002/fsn3.1896. 33282258 PMC7684621

[ref49] Yassaroh Y.; Woortman A. J. J.; Loos K. A new way to improve physicochemical properties of potato starch. Carbohydr. Polym. 2019, 204, 1–8. 10.1016/j.carbpol.2018.09.082. 30366520

[ref50] Lu X. X.; Luo Z. G.; Yu S. J.; Fu X. Lipase-catalyzed Synthesis of Starch Palmitate in Mixed Ionic Liquids. J. Agric. Food Chem. 2012, 60 (36), 9273–9279. 10.1021/jf303096c. 22920292

[ref51] Cai S. W.; Gu S. Y.; Li X.; Wan S. H.; Chen S. B.; He X. R. Controlled grafting modification of starch and UCST-type thermosensitive behavior in water. Colloid Polym. Sci. 2020, 298 (8), 1053–1061. 10.1007/s00396-020-04670-z.

[ref52] Seantier B.; Bendahou D.; Bendahou A.; Grohens Y.; Kaddami H. Multi-scale cellulose based new bio-aerogel composites with thermal super-insulating and tunable mechanical properties. Carbohydr. Polym. 2016, 138, 335–348. 10.1016/j.carbpol.2015.11.032. 26794770

[ref53] Wu K.; Fang Y.; Wu H. X.; Wan Y.; Qian H.; Jiang F. T.; Chen S. Improving konjac glucomannan-based aerogels filtration properties by combining aerogel pieces in series with different pore size distributions. Int. J. Biol. Macromol. 2021, 166, 1499–1507. 10.1016/j.ijbiomac.2020.11.029. 33181223

[ref54] Ding L.; Chen L. Y.; Hu L. C.; Feng X. L.; Mao Z. P.; Xu H.; Wang B. J.; Sui X. F. Self-healing and acidochromic polyvinyl alcohol hydrogel reinforced by regenerated cellulose. Carbohydr. Polym. 2021, 255, 117331 10.1016/j.carbpol.2020.117331. 33436174

[ref55] Ciric J.; Loos K. Synthesis of branched polysaccharides with tunable degree of branching. Carbohydr. Polym. 2013, 93 (1), 31–37. 10.1016/j.carbpol.2012.04.008. 23465898

[ref56] Qiao D. L.; Yu L.; Bao X. Y.; Zhang B. J.; Jiang F. T. Understanding the microstructure and absorption rate of starch-based superabsorbent polymers prepared under high starch concentration. Carbohydr. Polym. 2017, 175, 141–148. 10.1016/j.carbpol.2017.07.071. 28917849

[ref57] Pourjavadi A.; Ghasemzadeh H.; Soleyman R. Synthesis, characterization, and swelling behavior of alginate-g-poly(sodium acrylate)/kaolin superabsorbent hydrogel composites. J. Appl. Polym. Sci. 2007, 105 (5), 2631–2639. 10.1002/app.26345.

[ref58] Konieczny J.; Loos K. Facile Esterification of Degraded and Non-Degraded Starch. Macromol. Chem. Phys. 2018, 219 (18), 6 10.1002/macp.201800231.

[ref59] Xiao X. M.; Yu L.; Xie F. W.; Bao X. Y.; Liu H. S.; Ji Z. L.; Chen L. One-step method to prepare starch-based superabsorbent polymer for slow release of fertilizer. Chem. Eng. J. 2017, 309, 607–616. 10.1016/j.cej.2016.10.101.

[ref60] Zou W.; Liu X. X.; Yu L.; Qiao D. L.; Chen L.; Liu H. S.; Zhang N. Z. Synthesis and Characterization of Biodegradable Starch-Polyacrylamide Graft Copolymers Using Starches with Different Microstructures. J. Polym. Environ. 2013, 21 (2), 359–365. 10.1007/s10924-012-0473-y.

[ref61] Ahmadi-Abhari S.; Woortman A. J. J.; Hamer R. J.; Loos K. Rheological properties of wheat starch influenced by amylose-lysophosphatidylcholine complexation at different gelation phases. Carbohydr. Polym. 2015, 122, 197–201. 10.1016/j.carbpol.2014.12.063. 25817659

[ref62] Deng C. C.; Brooks W. L. A.; Abboud K. A.; Sumerlin B. S. Boronic Acid-Based Hydrogels Undergo Self-Healing at Neutral and Acidic pH. ACS Macro Lett. 2015, 4 (2), 220–224. 10.1021/acsmacrolett.5b00018. 35596411

[ref63] Ding Q. Q.; Xu X. W.; Yue Y. Y.; Mei C. T.; Huang C. B.; Jiang S. H.; Wu Q. L.; Han J. Q. Nanocellulose-Mediated Electroconductive Self-Healing Hydrogels with High Strength, Plasticity, Viscoelasticity, Stretchability, and Biocompatibility toward Multifunctional Applications. ACS Appl. Mater. Interfaces 2018, 10 (33), 27987–28002. 10.1021/acsami.8b09656. 30043614

[ref64] Song L. Z.; Zhu T. Y.; Yuan L.; Zhou J. J.; Zhang Y. Q.; Wang Z. K.; Tang C. B. Ultra-strong long-chain polyamide elastomers with programmable supramolecular interactions and oriented crystalline microstructures. Nat. Commun. 2019, 10, 1315 10.1038/s41467-019-09218-6. 30899014 PMC6428834

